# Overexpression of cathepsin S exacerbates lupus pathogenesis through upregulation TLR7 and IFN-α in transgenic mice

**DOI:** 10.1038/s41598-021-94855-5

**Published:** 2021-08-11

**Authors:** Jinhee Lee, Soyoung Jang, Minjee Choi, Mincheol Kang, Su-Geun Lim, SI-Yong Kim, Soyeon Jang, Jiwon Ko, Eungyung Kim, Junkoo Yi, Yeonsik Choo, Myoung Ok Kim, Zae Young Ryoo

**Affiliations:** 1 School of Life Sciences, BK21 FOUR KNU Creative BioResearch Group, Kyungpook National University, Daegu, 41566 Korea; 2 Core Protein Resources Center, DGIST, Daegu, Republic of Korea; 3 Department of Physiology and Cell Biology, School of Medicine, University of Nevada Reno, Reno, NV 89557 USA; 4 Gyeongsangbukdo Livestock Research Institute, Yeongju, Republic of Korea; 5 Department of Biology, Kyungpook National University, Daegu, South Korea; 6 Department of Animal Science and Biotechnology, Kyungpook National University, Sangju-si, Gyeongsangbuk-do 37224 Republic of Korea

**Keywords:** Biochemistry, Immunology, Molecular biology

## Abstract

Systemic lupus erythematosus (SLE) is a chronic autoimmune disease that affects multiple organs. Recent studies suggest relevance between cysteine protease cathepsin S (CTSS) expression and SLE. To investigate the mechanism of CTSS in SLE, CTSS-overexpressing transgenic (TG) mice were generated, and induced lupus-like symptoms. Eight months later, the TG mice spontaneously developed typical SLE symptoms regardless of the inducement. Furthermore, we observed increased toll-like receptor 7 (TLR7) expression with increased monocyte and neutrophil populations in the TG mice. In conclusion, overexpression of CTSS in mice influences TLR7 expression, autoantibodies and IFN-α, which leads to an autoimmune reaction and exacerbates lupus-like symptoms.

## Introduction

Systemic lupus erythematosus (SLE) is a chronic inflammatory disease commonly observed in women^1,2^. The deposition of immune complexes, in which autoantibodies against nucleic acid–protein complexes accumulate in target tissues including skin, kidneys, heart, lungs, musculoskeletal system, hematopoietic organs, and even the central nervous system is characteristic of this disease^1–3^.

Several cytokines, including interferon-α (IFN-α), TNF-α, IL-17 and IFN-γ, have been implicated in SLE pathogenesis^4^. Of them, IFN-α is linked with the active disease, amount of autoantibodies and incidence of renal involvement in SLE^5–7^. Moreover, it activates monocytes to produce the chemoattractants CCL2, CCL7 and CCL12, which collectively recruit monocytes to inflammatory regions^8^.

Toll-like receptor 7 (TLR7) is a pattern recognition receptor that highly expressed in SLE patients and is mainly located in the endosomes of various immune cells^9^. In SLE, excess apoptosis, tissue damage and decreased clearance of apoptotic bodies lead to the accumulation of immunogenic self-nucleic acid-containing particles^10^. Immune cells with TLR7 recognize this self-nucleic acid complex as a ligand and produce INF-α.

To investigate the SLE in the murine model, the natural hydrocarbon oil obtained from shark liver named 2,6,10,14-tetramethylpentadecane (TMPD, commonly known as pristane) is used^11,12^. It stimulates the secretion of IFN-α through monocytes and leads to indirect activation of TLR7^13^. Pristane-treated mice develop chronic inflammation and present symptoms similar to human SLE, including arthritis, diverse autoantibody production, glomerulonephritis and pulmonary vasculitis^14,15^.

Cathepsin S (CTSS), which belong to cathepsin family, is mainly active at acidic pH and typically active in lysosomes. However, Since CTSS can function within a broad pH range of 6.0–7.5, it remains stable and has a physiological role both inside and outside of lysosomes and even in the extracellular environment^16^. CTSS plays a critical role in antigen presentation, attributed to expression of major histocompatibility complex (MHC) class II. Further, CTSS cleaves various proteins and components of the basal membrane^17,18^. Due to these activities, CTSS production can lead to immune cell infiltration into peripheral vascular regions, triggering a vigorous immune response^19–23^.

It has been shown that CTSS expression is increased in the plasma of SLE patients compared with healthy controls^24^. Furthermore, injection of a CTSS antagonist suppresses SLE and prevents lupus nephritis by inhibiting MHC class II-mediated T and B cell priming, germinal centre formation and B cell maturation into plasma cells^25^. In addition, CTSS promotes PAR-2-driven endothelial injury in organs affected by autoimmune tissue inflammation^24^.

In the present study, we investigated the function of CTSS in SLE pathogenesis. We observed spontaneous lupus pathology in the skin and kidneys of 8-month-old transgenic (TG) mice. These mice showed increased monocyte levels and IFN-α cytokine expression with increased TLR7 expression, glomerulonephritis and increased infiltration of monocytes and IFN-α cytokines. These symptoms were exacerbated by pristane injection. Therefore, this study is the first to reveal that CTSS is involved in the pathogenesis of SLE via upregulation of TLR7–IFN-α pathway.

## Results

### Increased inflammatory response in 8-month-old CTSS TG mice

**Figure 1:**
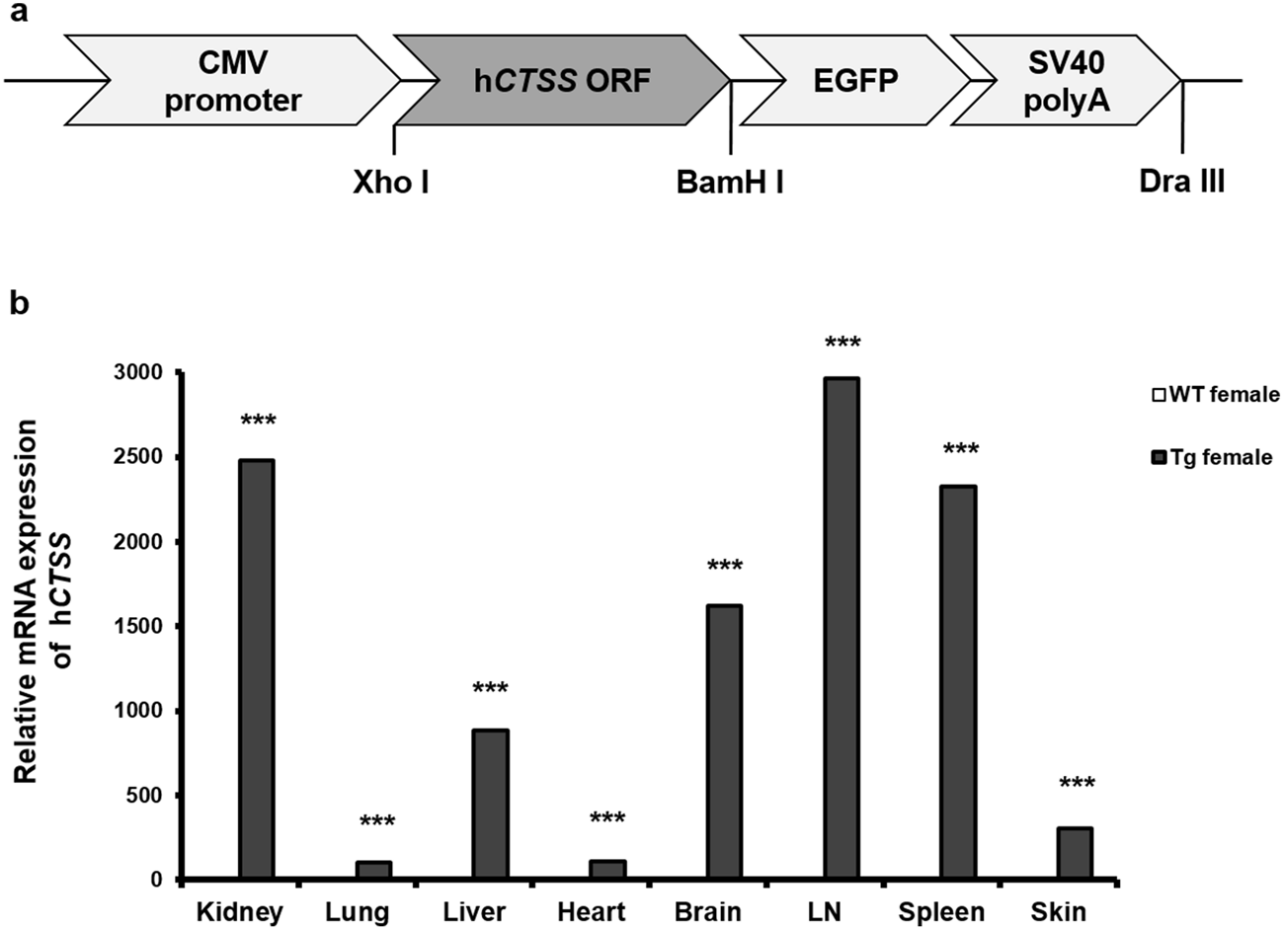
Cathepsin S (CTSS) overexpression vector construction and transgene expression in various tissues. (**a**) Schematic diagram of transgene vector construction for generation of CTSS-overexpressing TG mice. A cytomegalovirus (CMV) promoter was used to overexpress CTSS in all organs. *EGFP* enhanced green fluorescent protein, *ORF* open reading frame. (**b**) Human CTSS mRNA expression in various organs of 10-week-old WT and TG female mice measured by qRT-PCR. *TG* transgenic, *WT* wild type, *LN* lymph node. ***p < 0.001 versus untreated WT group.

CTSS-overexpressing TG mice were generated by transfection with a cytomegalovirus promoter, which mediates the expression of protein throughout the entire body (Fig. 1a). The expression of human CTSS is exceptionally high in all organs of TG female mice in comparison with wild type (WT) female mice. Notably, the mRNA level of CTSS was significantly high in the kidneys, lymph nodes, spleen and skin, a condition known as organ-affected SLE (Fig. 1b).

**Figure 2:**
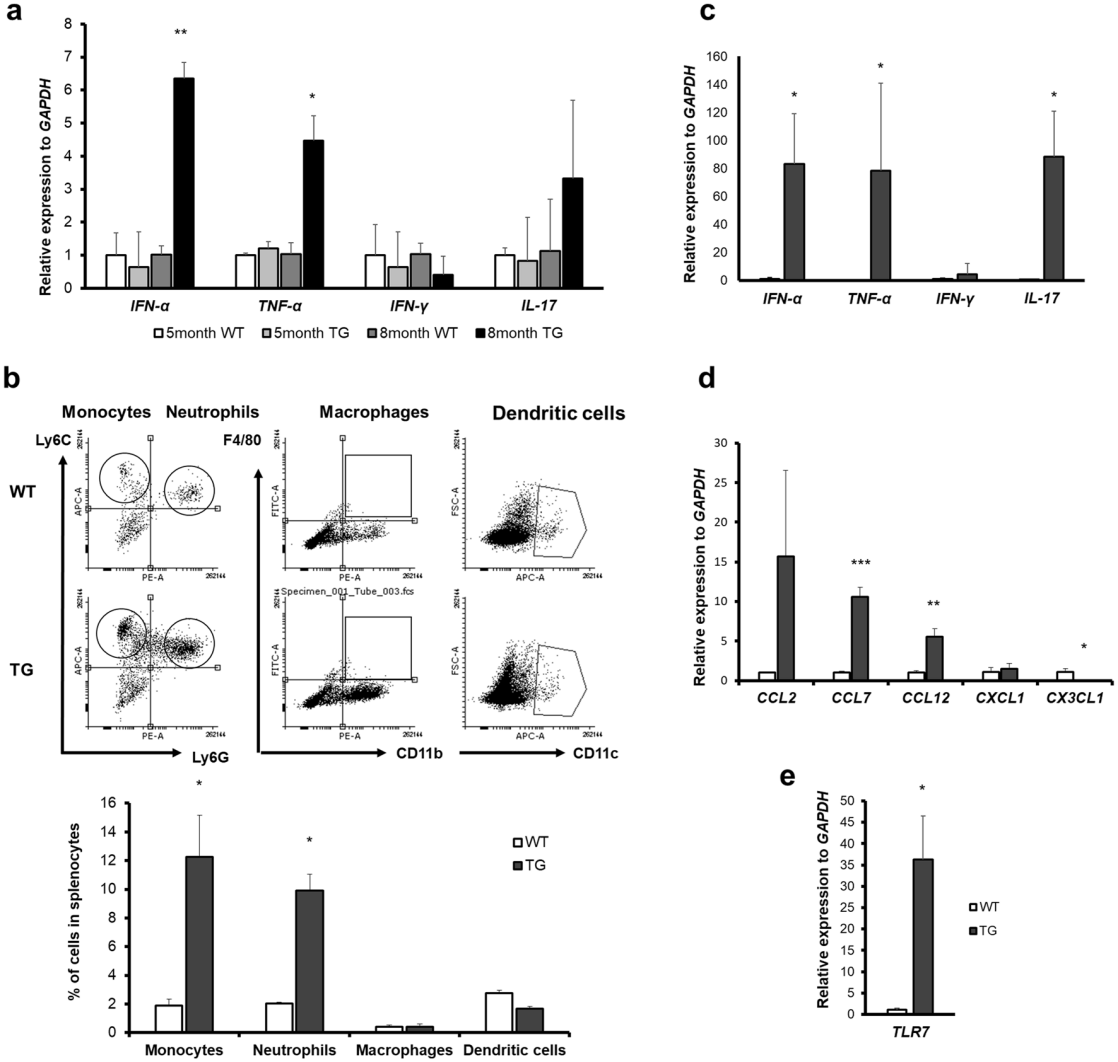
Generation of spontaneous inflammation in 8-month-old transgenic (TG) mice. (**a**) mRNA levels of cytokines (*IFN-α*, *TNF-α*, *IL-17* and *IFN-γ*) in the spleens of 5- and 8-month-old WT and TG mice measured by qRT-PCR. (**b**) Flow cytometric analysis of single cells from the spleens of 8-month-old mice. ((**b**), upper) A strategy to determine the population of monocytes, neutrophils, macrophages and dendritic cells is represented. To investigate the population of monocytes and neutrophils, CD11b^+^ cells were gated first. Ly6C^+^Ly6G^+^ cells were considered to be neutrophils, and Ly6C^+^Ly6G^-^ cells were considered to be monocytes. CD11b^+^F4/80^+^ cells served as macrophages, and CD11c^+^ cells represented neutrophils. ((**b**), lower) Monocyte, neutrophil, macrophage and dendritic cell populations are represented. (**c**) mRNA levels of cytokines *IFN-α*, *TNF-α*, *IL-17* and *IFN-γ*, (**d**) mRNA expression levels of chemokines *CCL2*, *CCL7*, *CCL12*, *CXCL1* and *CX3CL1*. (**e**) TLR7 mRNA expression levels in the kidneys of 8-month-old WT and TG mice, measured by qRT-PCR. WT, wild type. *p < 0.05; **p < 0.01, ***p < 0.001 versus WT group of the same age.

Whether chronically increased CTSS generates spontaneous lupus pathogenesis, we examined the 8-month-old WT and TG mice. In spleen, a significantly increased level of *IFN-α* and *TNF-α* mRNA was detected in TG mice compared to other groups. However, *IFN-γ* and *IL-17* mRNA expression in the TG mice were not significantly increased (Fig. 2a). Furthermore, we measured the immune cell population in the spleens via FACS. We observed that CD11b^+^Ly6c^+^Ly6G^-^ monocytes and CD11b^+^Ly6c^+^Ly6G^+^ neutrophils were significantly increased in TG mice compared with WT mice. The populations of other immune cells, CD11b^+^F4/80^+^ macrophages and CD11c^+^ dendritic cells did not differ between WT and TG mice (Fig. 2b).

Next, the kidney, a representative organ that is damaged by the accumulation of immune complexes in SLE, is examined. The mRNA expression of *IFN-α*, *TNF-α* and *IL-17* inflammatory cytokines was increased significantly in the kidneys of TG mice relative to those of WT mice (Fig. 2c). In addition, expression of chemokines, the monocytes attractants activated by IFN-α, was significantly higher in TG mice than WT mice. *CXCL1* and *CX3CL1* are representative chemokines that attract neutrophils and macrophages, respectively. *CXCL1* chemokine expression was not different between WT and TG mice; *CX3CL1* expression was significantly decreased in TG mice compared with WT mice (Fig. 2d). Furthermore, TLR7 mRNA expression was increased TG mice (Fig. 2E). Therefore, it is assumed that increased CTSS activates TLR7 and IFN*-*α signaling–mediated inflammation TG mice.

### Spontaneous inflammation in CTSS TG mice and deterioration due to pristane injection

**Figure 3:**
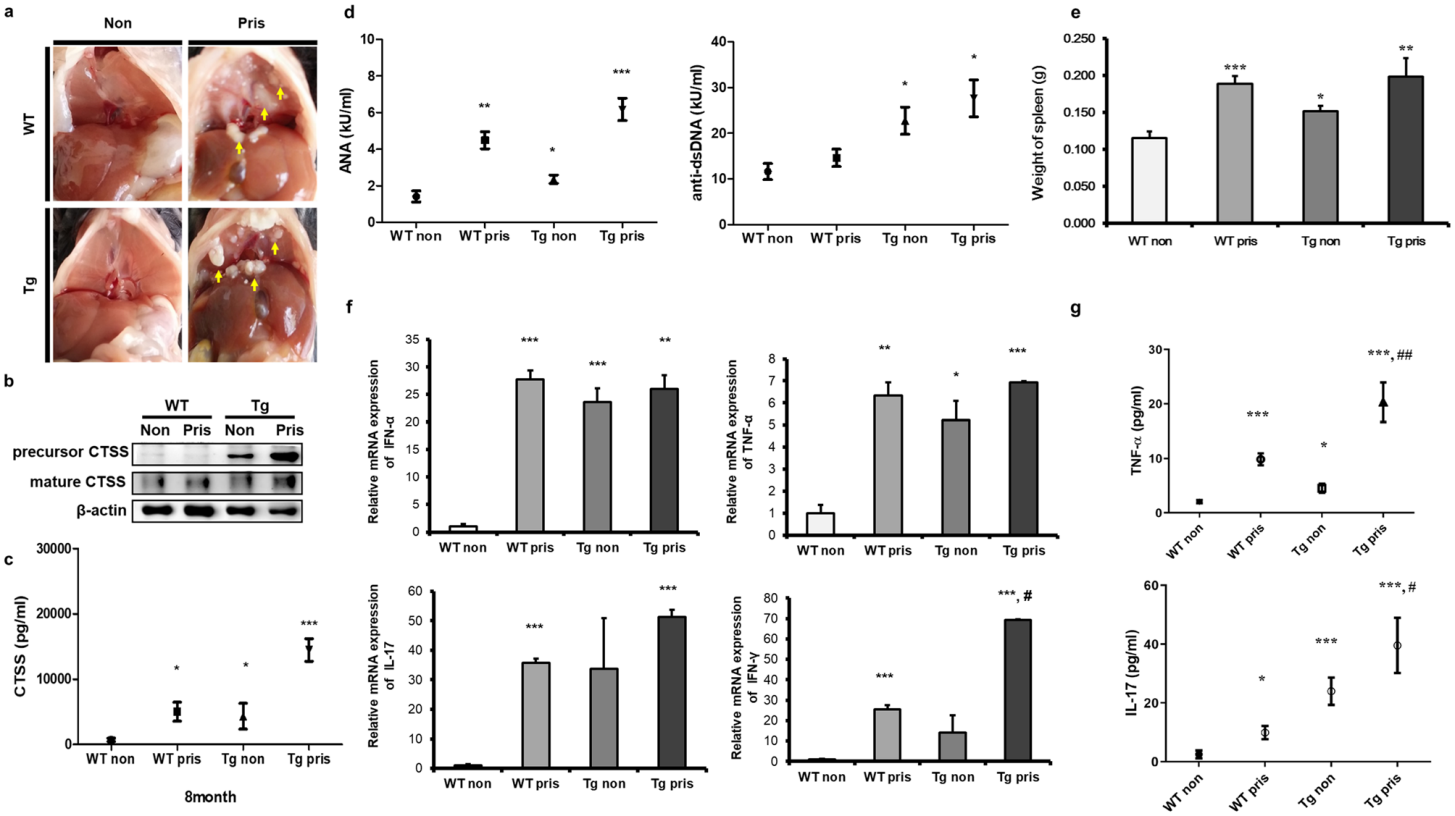
Increased cathepsin S (CTSS) expression induced by pristane injection with systemic lupus-like symptoms. (**a**) Lipogranuloma attached to the diaphragm (arrows) in WT and TG mice 8 months after pristane treatment. (**b**) Representative western blot showing CTSS expression in the spleen. β-actin was used as a loading control. Each band was cropped from same gel and grouped together. Original blots are shown in Supplementary Information. (**c**) Serum CTSS levels in WT and TG mice 8 months after treatment with or without pristane injection were measured by ELISA. *Non* pristane non-injected group, *Pris* pristane-injected group. (**d**) Serum ANAs and anti-dsDNA levels in WT and TG mice 8 months after treatment with pristane, as determined by ELISA. (**e**) Spleen weights of WT and TG mice treated with pristane. (**f**) *IFN-α*, *TNF-α*, *IL-17* and *IFN-γ* cytokine mRNA levels in spleens from WT and TG mice measured by qRT-PCR. (**g**) Concentrations of TNF-α and IL-17 in sera, as determined by ELISA. *Non* pristane non-injected group, *Pris* pristane-injected group. *WT* wild type, *TG* transgenic. *p < 0.05; **p < 0.01; ***p < 0.001, versus WT non-treated group, ^#^*p* < 0.05; ^##^*p* < 0.001, versus WT pristane-injected group.

To induce SLE, pristane was injected to the mice. After 8 months, the non-injected TG mice showed spontaneous inflammation. WT and TG mice both showed lipogranuloma, a chronic inflammatory response to pristane^14^, on the diaphragm (Fig. 3a). CTSS was increased in the spleen and sera of TG mice and so does the pristane-injected WT and TG mice (Fig. 3b,c).

We found that more severe SLE phenotypes were associated with higher titers of total immunoglobulin (IgG). Also, we observed that autonomous antibodies in TG mice were increased significantly compared with WT mice. Following pristane injection, there was a greater increase in autoantibodies in TG mice than in WT mice (Fig. 3d). Recording of spleen weight confirmed an increased spleen size in all groups, except for the non-injected WT group (Fig. 3e).

The mRNA expression levels of the pro-inflammatory cytokines *IFN-α*, *TNF-α*, *IFN-γ* and *IL-17* were measured in the spleen tissue by quantitative real-time PCR (qRT-PCR), and the corresponding protein levels in the serum were deduced by ELISA. In pristane-injected WT mice, all of these cytokines were significantly increased compared with non-injected WT mice. *IFN-γ* mRNA expression was not increased in pristane-injected TG mice compared with non-injected WT mice. However, after pristane injection, a significant increase in cytokines was detected in injected TG mice compared to injected WT mice. In addition, TNF-α and IL-17 levels in TG mice were further intensified by pristane treatment (Fig. 3f,g). Therefore, pristane injection exacerbated cytokine secretion in TG mice relative to WT mice. These results suggest that increased CTSS is associated with an increased inflammatory response.

### Lupus-like pathology were exacerbated in CTSS-overexpressing TG mice

**Figure 4:**
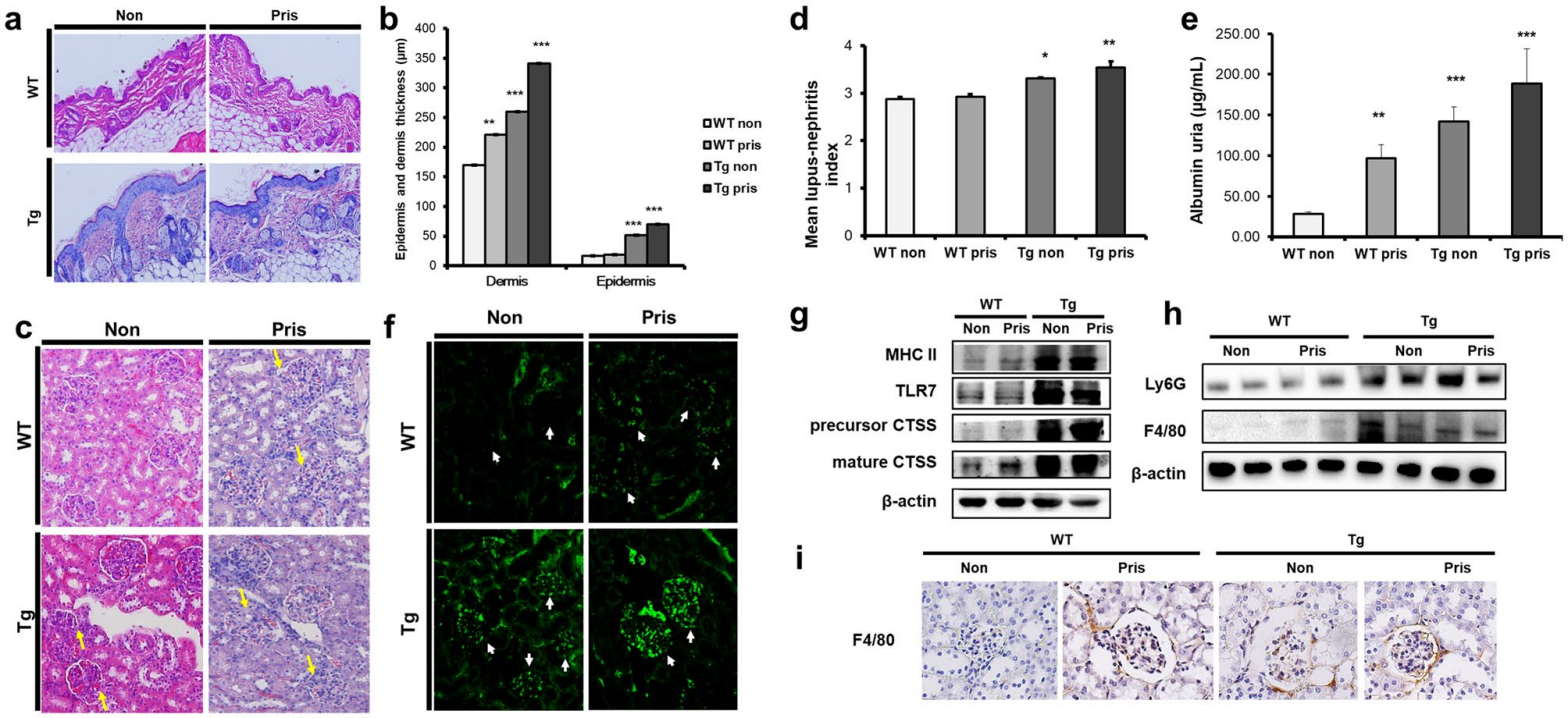
Histology of skin and renal pathology in pristane-injected cathepsin S (CTSS)—overexpressing mice. (**a**) H&E-stained section of dorsal skin (× 200 magnification) from WT and TG mice 8 months after treatment with pristane. (**b**) Epidermal and dermal thickness of dorsal skin from WT and TG mice. *WT* wild type, *TG* transgenic. **p < 0.01; ***p < 0.001, versus WT non-treated group. (**c**) H&E-stained kidney sections (× 200 magnification) from WT and TG mice 8 months after treatment with pristane. Arrows indicate glomerular abnormalities. (**d**) Renal histological scores represent nephritis severity in WT and TG mice 8 months after pristane injection. (**e**) Albumin concentration in urea measured by ELISA. (**f**) Glomerular immunoglobulin (IgG) and complement (C3) deposition analysed by direct immunofluorescence in WT and TG mice 8 months after treatment with pristane. Representative western blot showing, (**g**) MHC II, TLR7 and CTSS expression in the spleen and (**h**) expression of F4/80^+^ monocytes/macrophages and Ly6G^+^ neutrophils in the kidneys. β-actin was used as a loading control. Precursor and mature CTSS bands were cropped from different gel and grouped together. Other band were cropped from the different gel and grouped together. Original blots are shown in Supplementary Information (**i**) F4/80^+^ monocyte/macrophage infiltration analysed by immunohistochemistry (× 200 magnification) in WT and TG mice 8 months after treatment with pristane. *WT* wild type, *TG* transgenic. *p < 0.05; **p < 0.01; ***p < 0.001, versus untreated WT group.

SLE skin lesions represent dense superficial and deep perivascular and periadnexal lymphocytic infiltrates^26^. The epidermis and dermis were thicker in TG mice than in WT mice, regardless of pristane treatment (Fig. 4a). The thickness of the epidermis and dermis increased equally (Fig. 4b). Pristane-injected or non-injected TG mice presented more severe symptoms and immune cell infiltration, suggesting that epidermal and dermal thickening was characteristic of TG mice, and that CTSS can cause serious skin abnormalities. It also implies that TG mice could be used as an authentic animal model of SLE.

To assess the overall characteristics of the kidney glomeruli, hematoxylin–eosin (H&E) staining was conducted. In three groups, except non-injected WT mice, kidney abnormalities were detected, including hypercellularity in the glomerular capillaries and mesangial matrix, glomeruli with narrowing of the cavity, and mild peritubular mononuclear cell infiltration (Fig. 4c). The renal histopathological scores of the TG mice were significantly increased compared with those of WT mice, regardless of pristane injection (Fig. 4d).

To quantify urinary protein excretion, which is related to renal dysfunction, urine samples were collected from individual mice at 8 months after pristane injection and the albumin concentrations in the urine were examined by albumin ELISA (Fig. 4e).

Immunofluorescence staining was used to detect glomerular IgG and complement C3 deposition in glomerular sections from pristane-injected and non-injected WT and TG mice. In contrast to the other groups, TG pristane-treated mice showed IgG and C3 deposits within the glomeruli, suggesting that glomerulonephritis had already progressed. Additionally, the severity of IgG and C3 deposition in non-injected TG mice and pristane-injected WT mice was comparable (Fig. 4f). The TG pristane-treated mice presented more severe renal dysfunction and glomerulonephritis. Interestingly, untreated TG mice also presented these symptoms. These results suggest that the TG mice spontaneously developed renal disease over time.

### Increasing TLR7 and macrophage and neutrophil infiltration into the kidneys of CTSS TG mice

TLR7 is known to play a central role in the progression of SLE. Western blot analysis showed increased TLR7 expression in the spleens of TG mice compared with WT mice (Fig. 4g). We identified F4/80^+^ macrophage/monocyte and Ly6G^+^ neutrophil infiltration in kidney tissue by western blot and immunohistochemistry where SLE symptoms were observed. The results showed that the amount of monocytes/macrophage and neutrophil infiltration in the kidneys of untreated or pristane-treated TG mice was significantly increased compared with WT mice (Fig. 4h). High amounts of F4/80 + monocytes/macrophages were found in the periglomerular area in untreated and pristane-treated TG mice (Fig. 4i).

## Discussion

SLE is a systemic autoimmune disorder that affects multiple organs, including the skin, kidneys, heart, lungs, musculoskeletal system, haematopoietic organs and even the central nervous system^1, 2^. Despite various investigations, the mechanisms of lupus are not entirely understood, which makes treatment difficult. A relationship between CTSS and SLE involving MHC class II has been suggested; however, the specific mechanism of this relationship and the factors involved are not fully understood. The present study demonstrates that CTSS has a severe effect on lupus-like disease through upregulation of TLR7 and INF-α in CTSS-overexpressing TG mice^27–29^.

Administration of a single dose of pristane to WT mice is widely applied as a method of causing human lupus-like symptoms, such as the production of autoantibodies and cytokines, increasing type I IFN as a signature and causing glomerulonephritis^11,12,14^. To examine the effect of CTSS on lupus-like disease, we also injected TG mice with pristane. At 8 months after pristane injection, the TG mice presented excessive cellular and humoral autoimmunity in addition to renal disorders compared with WT mice. Furthermore, CTSS-overexpressing mice showed a marked increase in the production of IL-17, TNF-α, IFN-α and IFN-γ, which are known to be associated with lupus development, and the chemoattractants CCL2, CCL7 and CCLl12, which are activated by IFN-α to attract monocytes to inflammatory sites.

The autoantibody levels of anti-dsDNA and ANA were both significantly increased in TG mice than in WT mice treated with pristane^30^. The glomerulonephritis was also more severe in TG mice, as shown by the deposition of immunoglobulins and complements determined by immunofluorescence.

The lupus-like phenotypes were much more excessive in pristane-treated TG mice than in any other group studied in the present experiment, including non-treated TG mice. Interestingly, we observed that symptoms in untreated TG mice were often more severe than in pristane-treated WT mice. This finding indicates that CTSS alone is sufficient to cause the development of lupus-like symptoms, which are likely to deteriorate to lupus-like disease due to the unique characteristics of CTSS.

The involvement of CTSS in SLE pathogenesis, through activated MHC class II and PAR2, has already been studied^24,25^. We confirmed increased MHC class II and PAR2 in our TG mice both previously^31^ and in the present study (Fig. 4G). However, we report here for the first time that CTSS involvement in TLR7–IFN-α-mediated SLE pathogenesis leads to macrophage/monocyte and neutrophil infiltration. Therefore, the interaction between TLR7, PAR2 and MHC class II activation through the CTSS-mediated inflammatory response exacerbates SLE pathogenesis.

Overall, the present study confirms that CTSS-overexpressing TG mice are an ideal experimental model for lupus disease. This model will provide insights into the pathogenesis of SLE and, furthermore, will be useful for the development of therapeutic targets.

## Methods

### Vector construction and generation of transgenic mice

The generation of human CTSS TG mice from a C57BL/6J background has been described previously^1^. Briefly, the transgene construct was microinjected into embryos obtained from C57BL/6J female mice. Microinjected embryos were transferred into the oviducts of pseudopregnant female ICR mice. All animal experiments were carried out in accordance with the relevant guidelines and regulations for animal experimentation and approved by the Institutional Animal Care and Use Committee of Kyungpook National University (Daegu, South Korea). Additionally, all methods were performed in accordance with the relevant guidelines and regulations and the study was carried out in compliance with the ARRIVE guidelines.

### Reagents and experimental protocol

At 10 weeks of age, WT and TG mice were intraperitoneally injected with pristane (500 µL) (TMPD; Sigma–Aldrich, St. Louis, MO, USA). All remaining mice, that is, the untreated WT and TG mice, were used as controls under the same conditions. The experiment ended at 5 and 8 months after the first pristane administration. The spleens, kidneys, skin, sera and urea of the four groups of mice were removed or collected and assessed for the production of cytokines and other parameters that affect SLE^2^.

### RNA isolation and qRT-PCR

Total RNA was isolated from the spleens, kidneys and skin of TG and WT mice 8 months after treatment with or without pristane injection. qRT-PCR was performed using a Step One Plus™ PCR system (Applied Biosystems, CA, USA) based on SYBR Green fluorescence after hybridisation with cDNA. Each set of primers (see Supplementary Table S1) was used for qRT-PCR, and β-actin or GAPDH was used as an internal quantitative control.

### ELISA

We obtained the sera of pristane-treated or untreated WT and TG mouse blood samples collected from the retro-orbital sinus of each mouse after anaesthesia. The secreted protein levels of TNF-α, IL-17 and IFN-γ in the sera were quantified using commercially available ELISA kits (R&D Systems, MN, USA). CTSS expression levels in the sera samples were also confirmed using an ELISA kit (Cusabio, Wuhan, China).

To measure autoantibody production, we used commercial ELISA kits to detect the total IgG concentration of anti-dsDNA and ANA (Alpha Diagnostic International, TX, USA).

Urine samples were collected at 8 months after treatment with or without pristane. To confirm albuminuria, we determined the albumin concentration in the urine of each of the four groups using a commercial mouse albumin ELISA quantification kit (Abcam, Cambridge, UK).

### Western blotting

To measure protein expression levels, spleen and kidney samples were washed with phosphate-buffered saline and then homogenised in Pro-Prep solution (iNtRON Biotechnology). In order to efficiently use the limited samples and avoid the non-specific back ground, membranes were cut prior to hybridization with antibodies. Donkey anti-CTSS (C19, Santa Cruz Biotechnology, CA, USA), rat anti-MHC class II (IBL-5/22, Santa Cruz Biotechnology), rabbit anti-TLR7 (Abcam), rat anti-F4/80 (Cl:A3-1, Bio-Rad, CA, USA), rat anti-Gr1 (RB6-8C5, Bio-Rad) and mouse monoclonal ß-actin (C4, Santa Cruz Biotechnology) antibodies, followed by HRP-conjugated anti-goat IgG (Santa Cruz Biotechnology), anti-mouse IgG (Santa Cruz Biotechnology) and anti-rat IgG (Sigma), were used. Using a Davinch-Chemi™ enhanced chemiluminescence detection system (GE Healthcare, Munich, Germany), immunoreactivity was measured, and the staining intensity was quantified using ImageJ software (National Institute of Health).

### Immunofluorescence

Direct immunofluorescence assays were performed along according to a custom immunofluorescence protocol on paraffin-embedded kidney sections. Glomerular IgG and complement C3 deposition in sections of 6 µm thickness were assessed using Alexa Fluor 488-conjugated goat anti-mouse IgG (1:200), goat anti-mouse IgM (1:200) and fluorescein non-conjugated rat anti-mouse C3 (1:200), followed by incubation with Alexa Fluor 488-conjugated rabbit anti-rat IgG (Abcam). All images were acquired using LAS AF software (Leica Microsystems, https://www.leica-microsystems.com).

### Histological analysis

Kidney and skin tissues were fixed in 4% paraformaldehyde, embedded in paraffin and sectioned at 6 μm. Glomerular abnormalities in paraffin-embedded section slides were assessed by H&E staining. To examine the degree of infiltration by neutrophils and macrophages, kidney sections were incubated with antibodies to monocytes/macrophages using neutrophil markers (rat anti-F4/80 and Gr1; Bio-Rad) at 1:50 dilution. HRP-conjugated anti-rat IgG (Sigma) was used as the secondary antibody at 1:100 dilution. The negative control constituted the secondary antibodies without the primary antibodies. Staining was developed using 3′,3-diaminobenzidine tetrahydrochloride (Vector Laboratories, San Francisco, USA), after which the slides were counterstained with hematoxylin.

Immunohistochemistry analyses of skin were performed using the same protocol, and epidermal and dermal thicknesses were measured by LAS 4.4 software (Leica Microsystems).

### Flow cytometric analysis

Single-cell spleen suspensions obtained from WT and TG mice were prepared by treatment with red blood cell lysis solution (0.15 M NH_4_Cl and 0.1 mM Na_2_EDTA) for 30 min at room temperature. Fluorescein isothiocyanate (FITC)-conjugated anti-CD11b (M1/70, Biolegend, CA, USA), FITC-anti-CD4 (GK1.5, BD Biosciences, CA, USA), FITC-anti-F4/80 (BM8, Thermo Fisher, MA, USA), phycoerythrin (PE)-conjugated anti-CD11b (M1/70, Biolegend), PE-conjugated anti-Ly6G (RB6-8C5, Thermo Fisher), APC-anti-Ly6C (HK1.4, Thermo Fisher) and Alexa Fluor 700-anti-CD11c (N418, Thermo Fisher) antibodies were used. Isotype control antibodies were used optimally.

All samples were acquired and analysed using a FACSAria™ III flow cytometer (BD Biosciences) and a BD Accuri C6 flow cytometer (BD Biosciences).

### Statistical analysis

Data are expressed as the mean ± SD of at least three independent experiments. The significance of differences between groups was evaluated by the Student’s *t*-test at *p* < 0.05. All statistical calculations were performed using GraphPad Prism 5.0 software.

## Supplementary Information

Supplementary Information 1.

Supplementary Information 2.
