# Supplementary material for: Overexpression of cathepsin S exacerbates lupus pathogenesis through upregulation TLR7 and IFN-α in transgenic mice
Source: Sci Rep. 2021 Aug 11;11:16348. doi: 10.1038/s41598-021-94855-5 (PMC8357804; doi:10.1038/s41598-021-94855-5)
Supplement: Supplementary file 1 — Supplementary Information 1. [file 41598_2021_94855_MOESM1_ESM.docx]

| **Gene** | **Primer sequence** | |
| --- | --- | --- |
| mouse *β-actin* | Forward | : 5’-TCACCCACAGTFTGCCCATCTACG-3’ |
|  | Reverse | : 5’-CGAAGTCTAGAGCAACATAGCAC-3’ |
| mouse *GAPDH* | Forward | : 5’-ACCACAGTCCATGCCATCAC -3’ |
|  | Reverse | : 5’-TCCACCACCCTGTTGCTGTA -3’ |
| human *CTSS* | Forward | : 5’-CGGCTGGTTTGTGTGCTCTT-3’ |
|  | Reverse | : 5’-AGAGATGCCAGTGGTGATCCA-3’ |
| mouse *CTSS* | Forward | : 5’-AAGCGGTGTCTATGACGACCC-3’ |
|  | Reverse | : 5’-GAGTCCCATAGCCAACCACAAG-3’ |
| mouse *TNFα* | Forward | : 5’-TGTGCTCAGAGCTTTCAACAAC-3’ |
|  | Reverse | : 5’-GCCCATTTGAGTCCTTGATG-3’ |
| mouse *IFNγ* | Forward | : 5’-CAACAGCAAGGCGAAAAAGG-3’ |
|  | Reverse | : 5’-TGGTGGACCACTCGGATGA-3’ |
| mouse *IFNα* | Forward | : 5’-AAGGACAGGAAGGATTTTGGATT-3’ |
|  | Reverse | : 5’-GAGCCTTCTGGATCTGTTGGTT-3’ |
| mouse *IL-17A* | Forward | : 5’-ACCGCAATGAAGACCCTGAT-3’ |
|  | Reverse | : 5’-TCCCTCCGCATTGACACA-3’ |
| mouse *TLR7* | Forward | : 5’-ACCGCAATGAAGACCCTGAT-3’ |
|  | Reverse | : 5’-TCCCTCCGCATTGACACA-3’ |
| mouse *CCL2* | Forward | : 5’-ACCGCAATGAAGACCCTGAT-3’ |
|  | Reverse | : 5’-TCCCTCCGCATTGACACA-3’ |
| mouse *CCL7* | Forward | : 5’-GAT CTC TGC CAC GCT TCT GT-3’ |
|  | Reverse | : 5’-ATA GCC TCC TCG ACC CAC TT-3’ |
| mouse *CCL12* | Forward | : 5’-GTC CTC AGG TAT TGG CTG GA-3’ |
|  | Reverse | : 5’-CAC TGG CTG CTT GTG ATT CT-3’ |
| mouse C*XCL1* | Forward | : 5’-GCT GGG ATT CAC CTC AAG AA-3’ |
|  | Reverse | : 5’-TCT CCG TTA CTT GGG GAC AC-3’ |
| mouse C*X3CL1* | Forward | : 5’-CGC GTT CTT CCA TTT GTG TA-3’ |
|  | Reverse | : 5’-AGC TGA TAG CGG ATG AGC AA-3’ |

**Table 1. Primers used for real time quantitative PCR**
