# Supplementary material for: Overexpression of cathepsin S exacerbates lupus pathogenesis through upregulation TLR7 and IFN-α in transgenic mice
Source: Sci Rep. 2021 Aug 11;11:16348. doi: 10.1038/s41598-021-94855-5 (PMC8357804; doi:10.1038/s41598-021-94855-5)
Supplement: Supplementary file 2 — Supplementary Information 2. [file 41598_2021_94855_MOESM2_ESM.pdf]

Western blot raw data

Figure 3B

With boxes

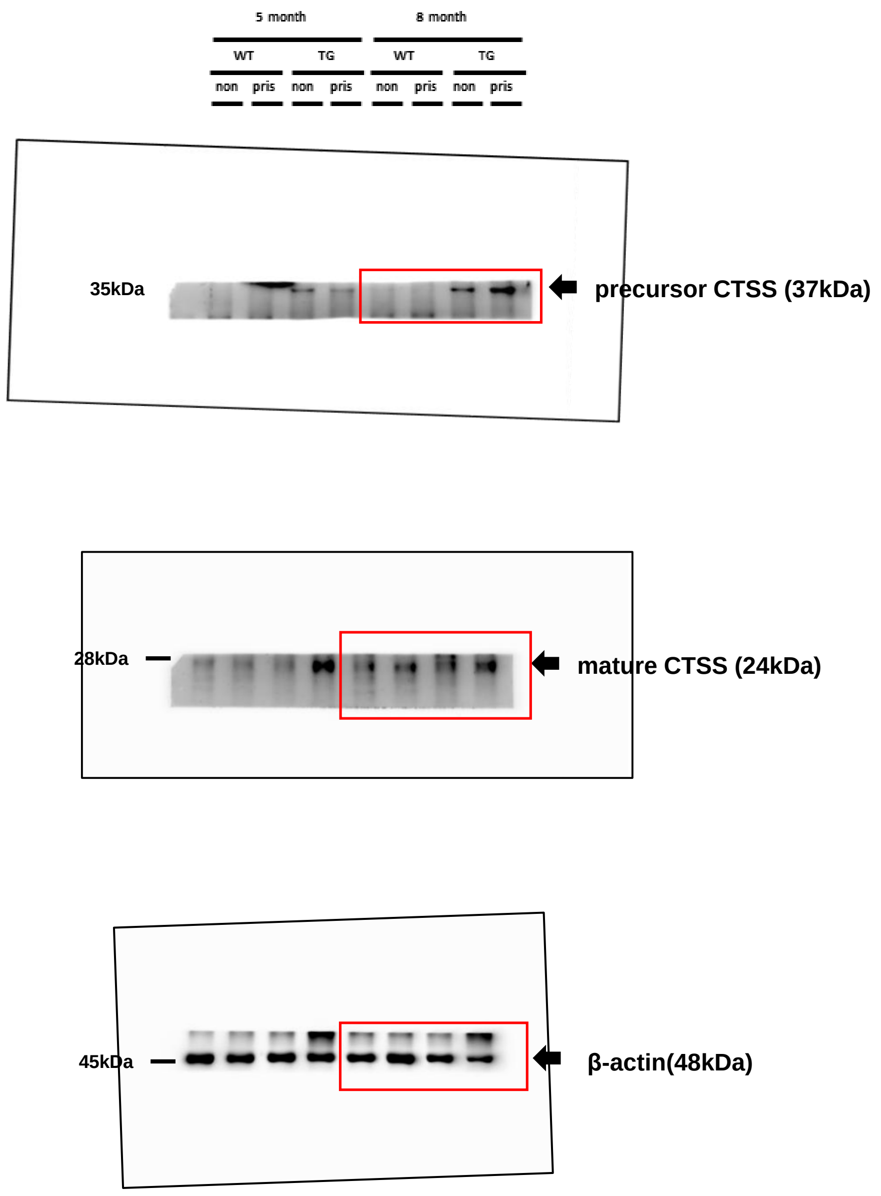

Without boxes

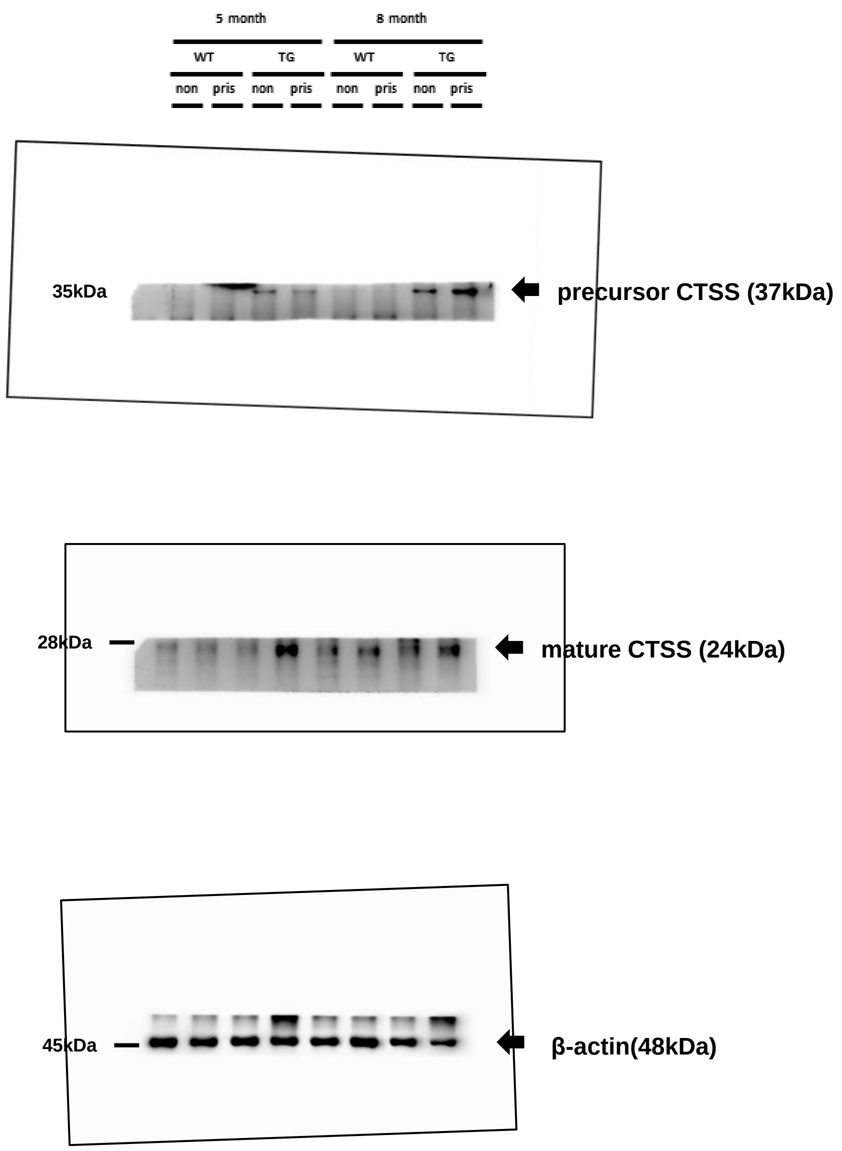

In order to efficiently use the limited samples and avoid the non-specific back ground, membranes were cut prior to hybridization with antibodies.

Western blot raw data

Repeat

Marker detection

Band detection

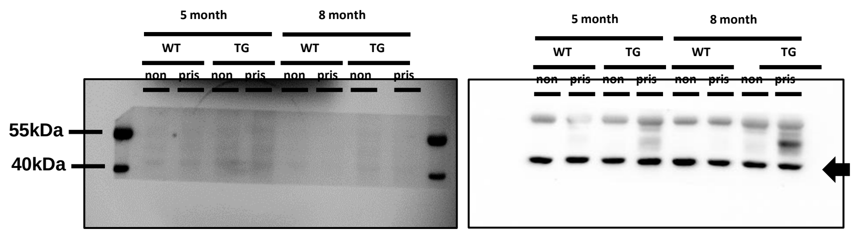

β-actin(48kDa)

CTSS (37, 24kDa)

precursor CTSS (37kDa)

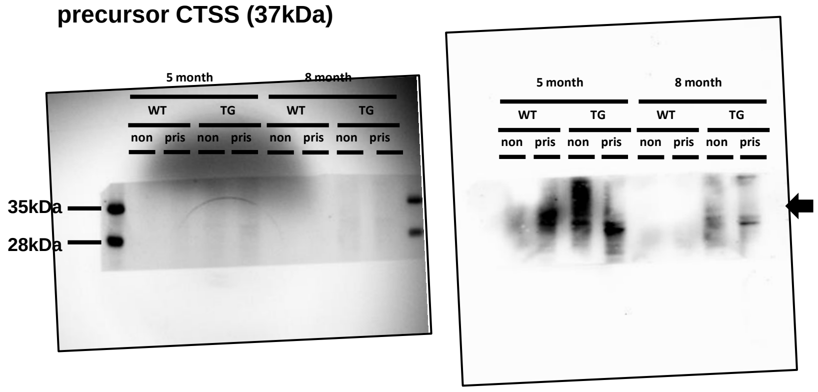

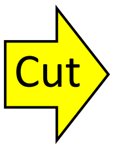

precursor CTSS (37kDa)

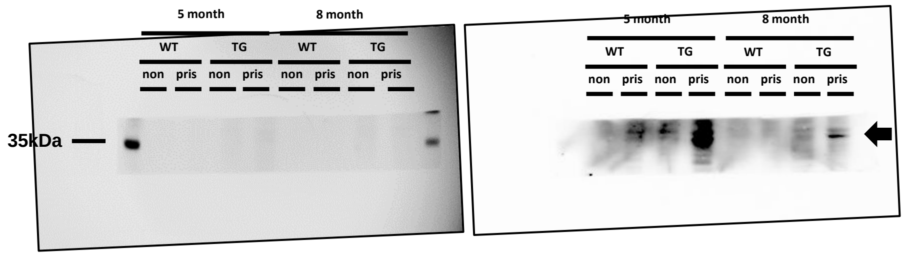

mature CTSS (24kDa)

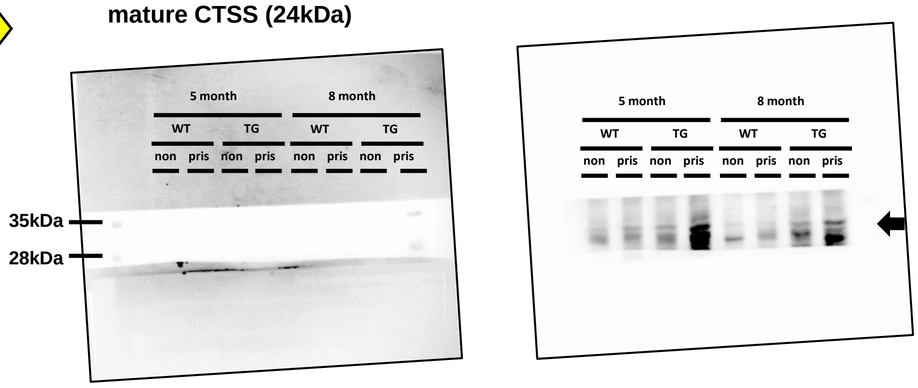

precursor CTSS (37kDa)

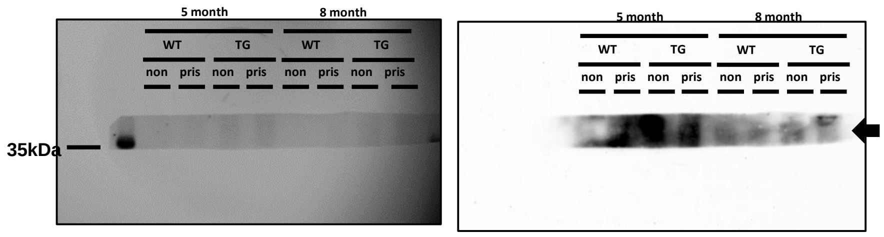

precursor CTSS (37kDa)

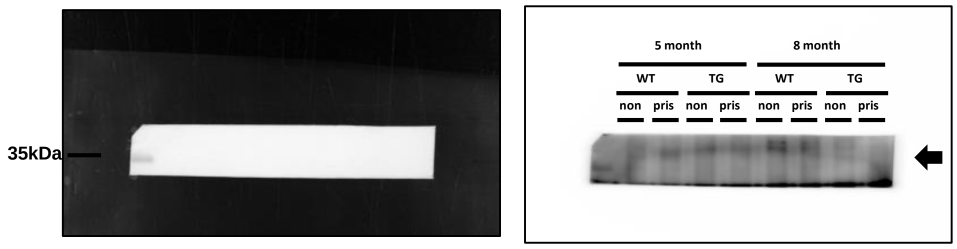

precursor CTSS (37kDa)

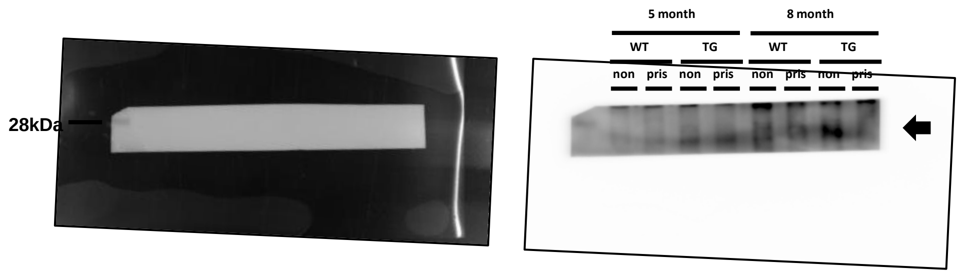

mature CTSS (24kDa)

β-actin(48kDa)

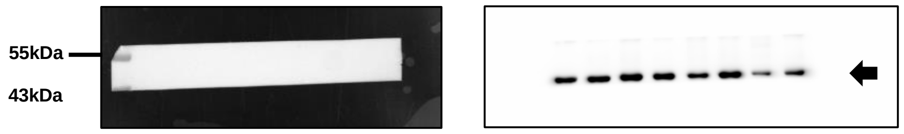

In order to efficiently use the limited samples and avoid the non-specific back ground, membranes were cut prior to hybridization with antibodies.

Western blot raw data

Figure 4G

With boxes

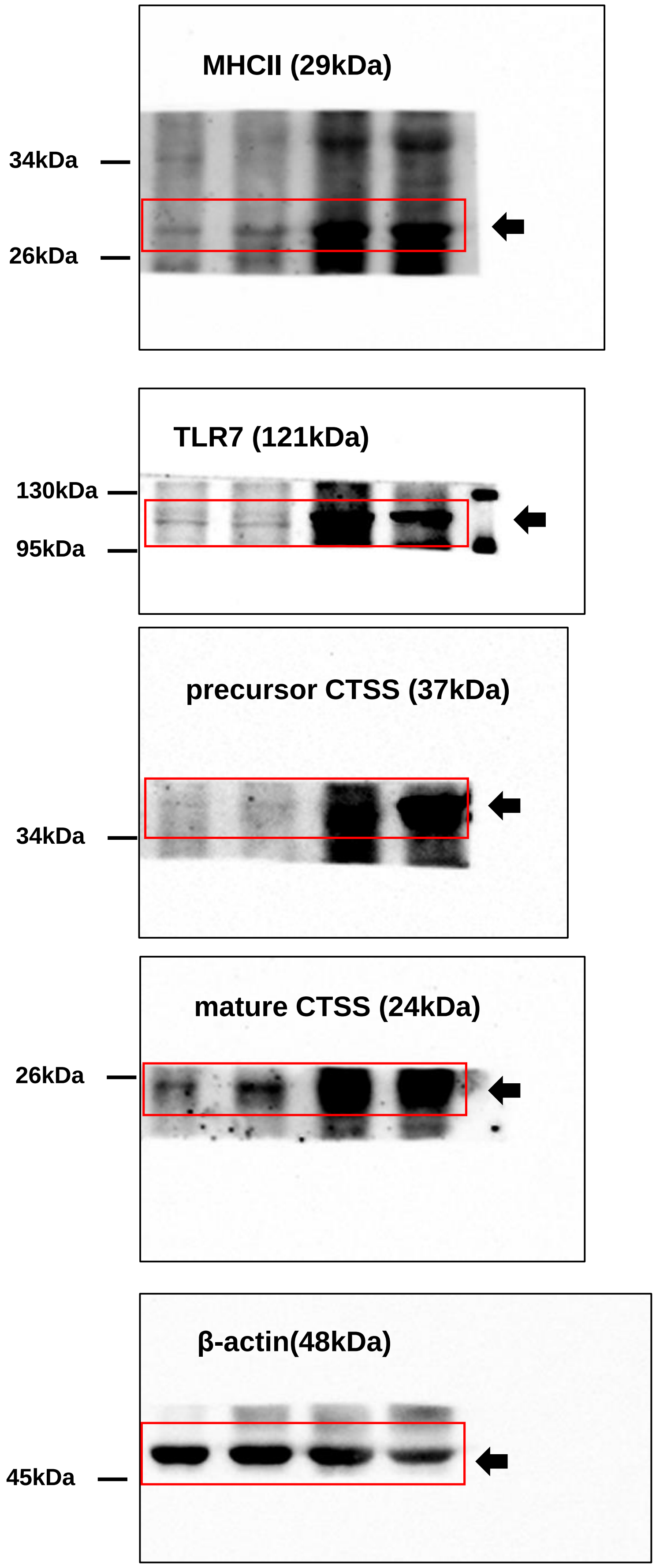

Without boxes

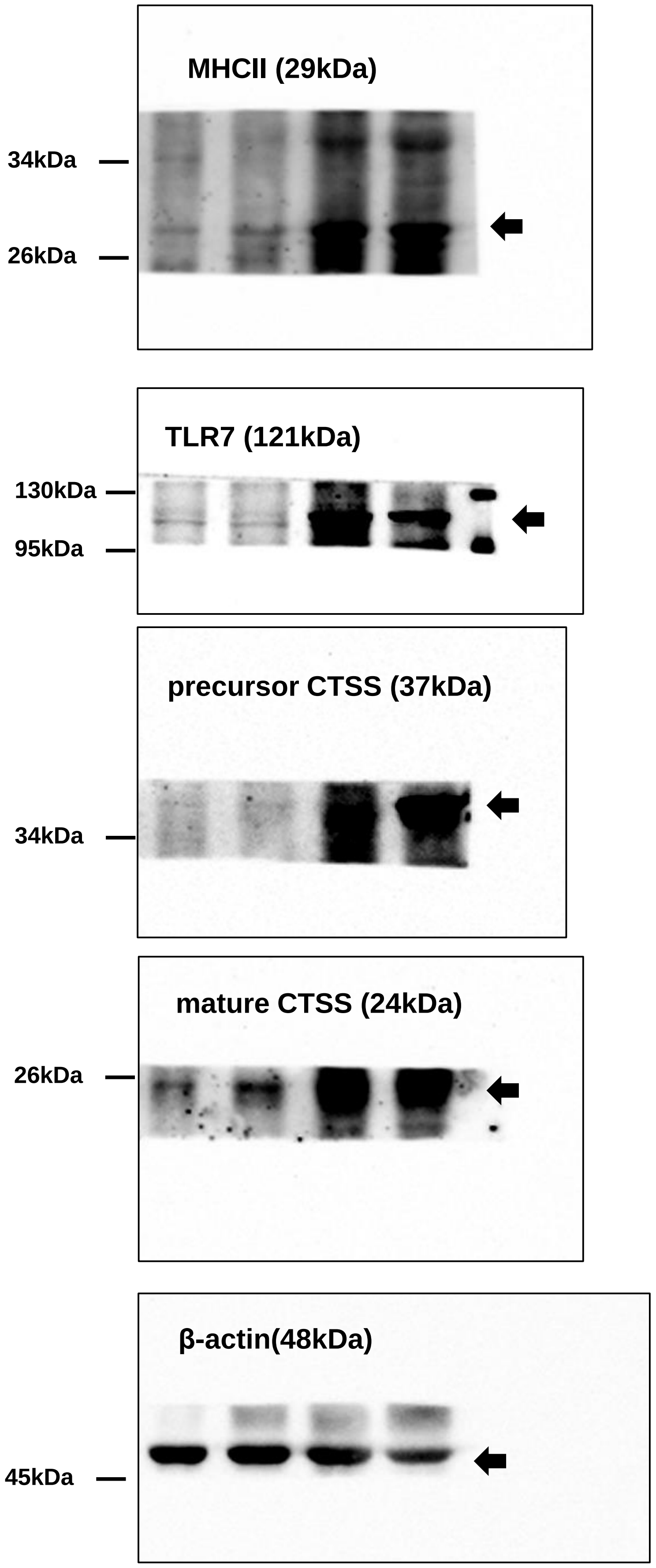

In order to efficiently use the limited samples and avoid the non-specific back ground, membranes were cut prior to hybridization with antibodies.

Western blot raw data

Repeat

MHCII (29kDa)

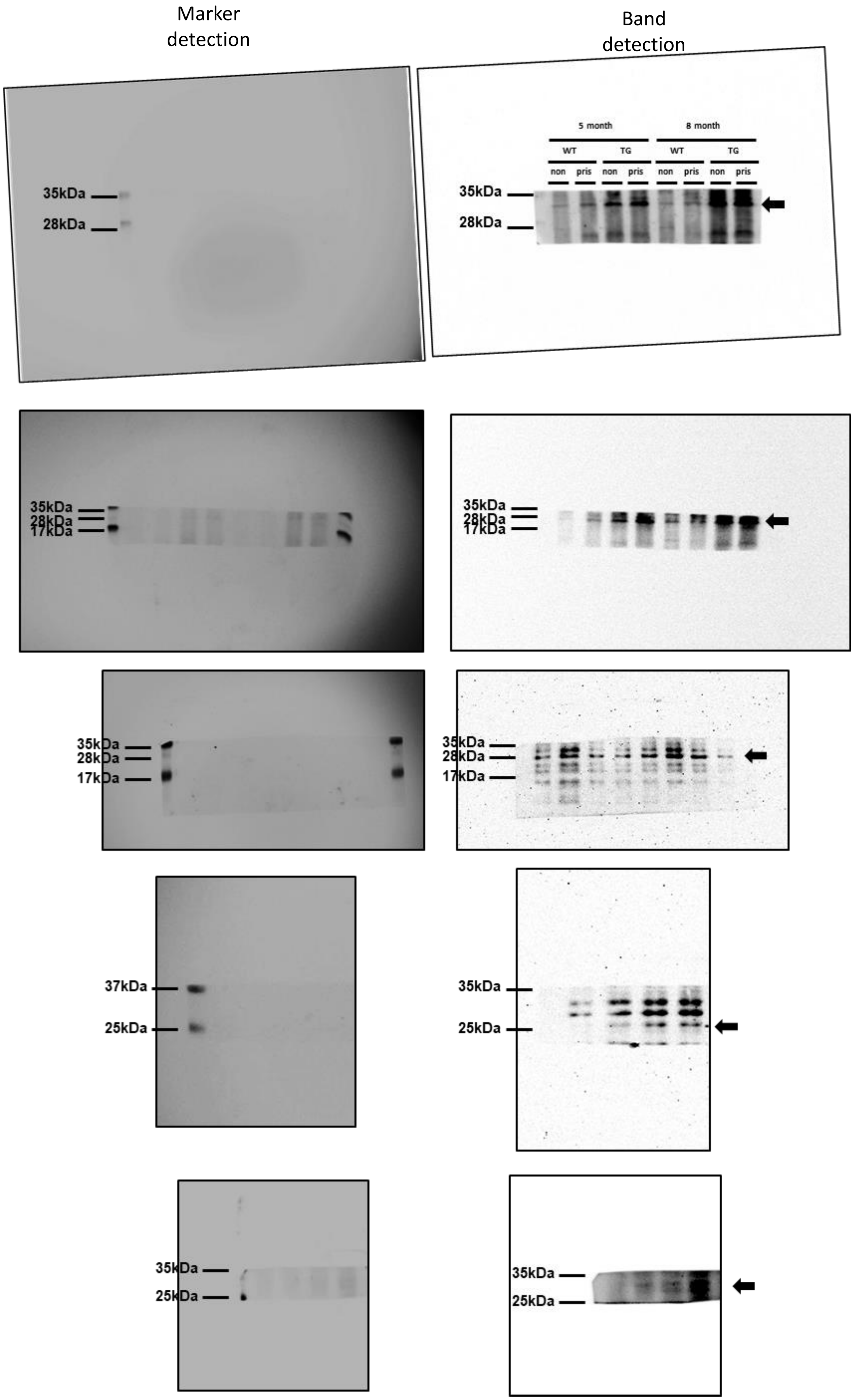

In order to efficiently use the limited samples and avoid the non-specific back ground, membranes were cut prior to hybridization with antibodies.

Western blot raw data

Repeat

CTSS (37,24kda)

Marker detection

Band detection

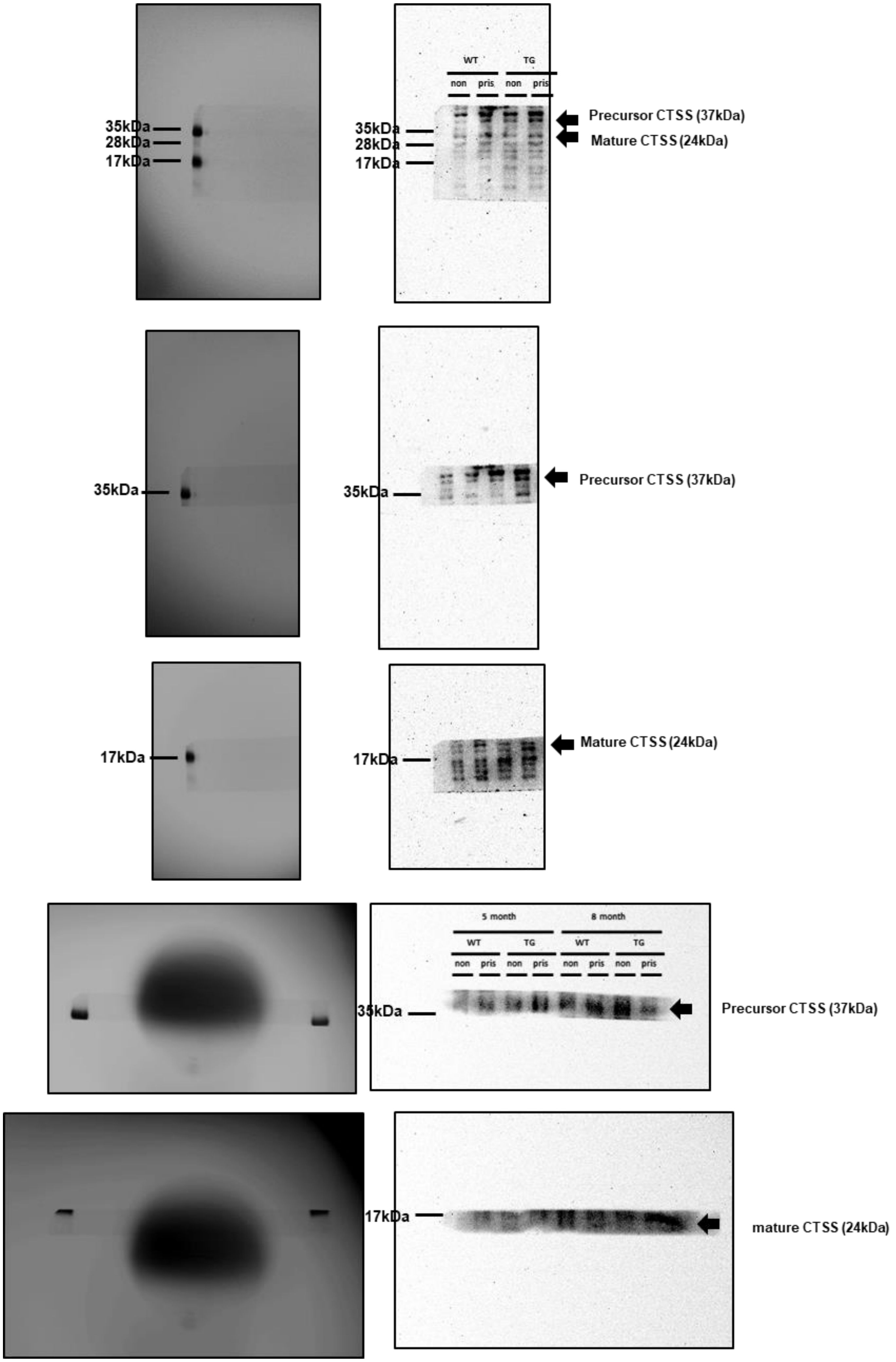

In order to efficiently use the limited samples and avoid the non-specific back ground, membranes were cut prior to hybridization with antibodies.

Western blot raw data

Repeat

TLR7 (121kDa)

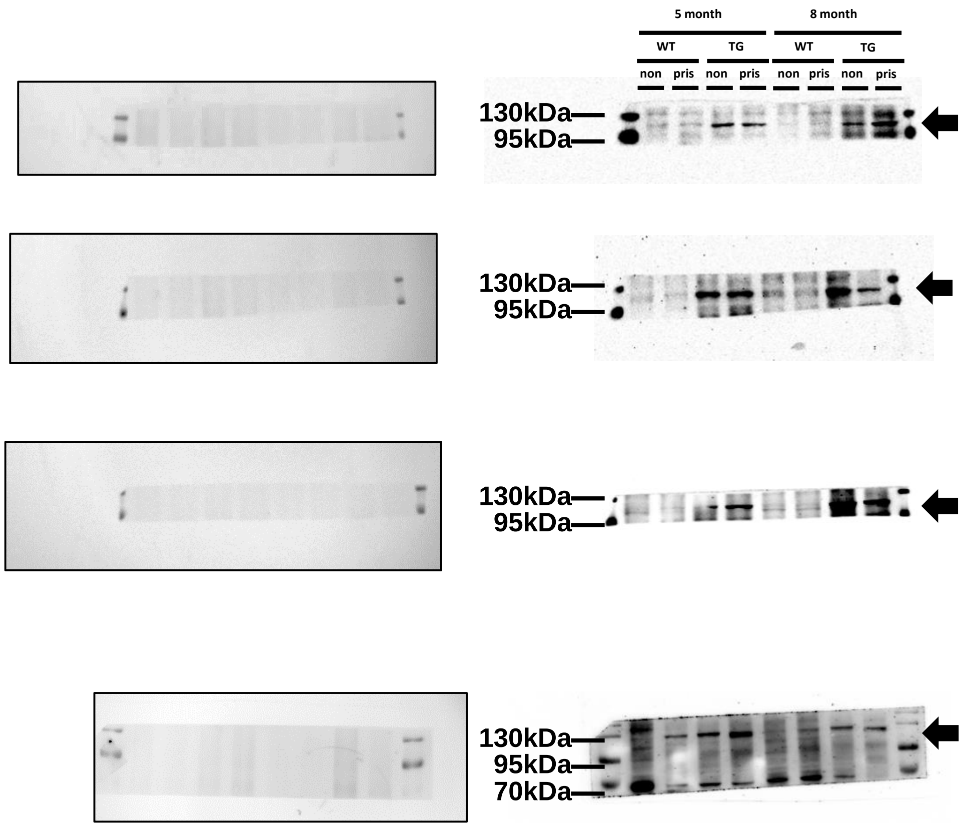

β -actin (48kDa)

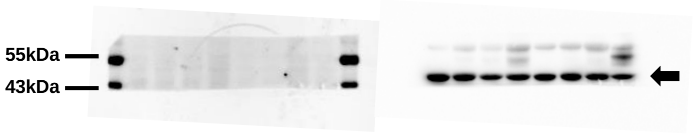

In order to efficiently use the limited samples and avoid the non-specific back ground, membranes were cut prior to hybridization with antibodies.

Western blot raw data

Figure 4H

With boxes

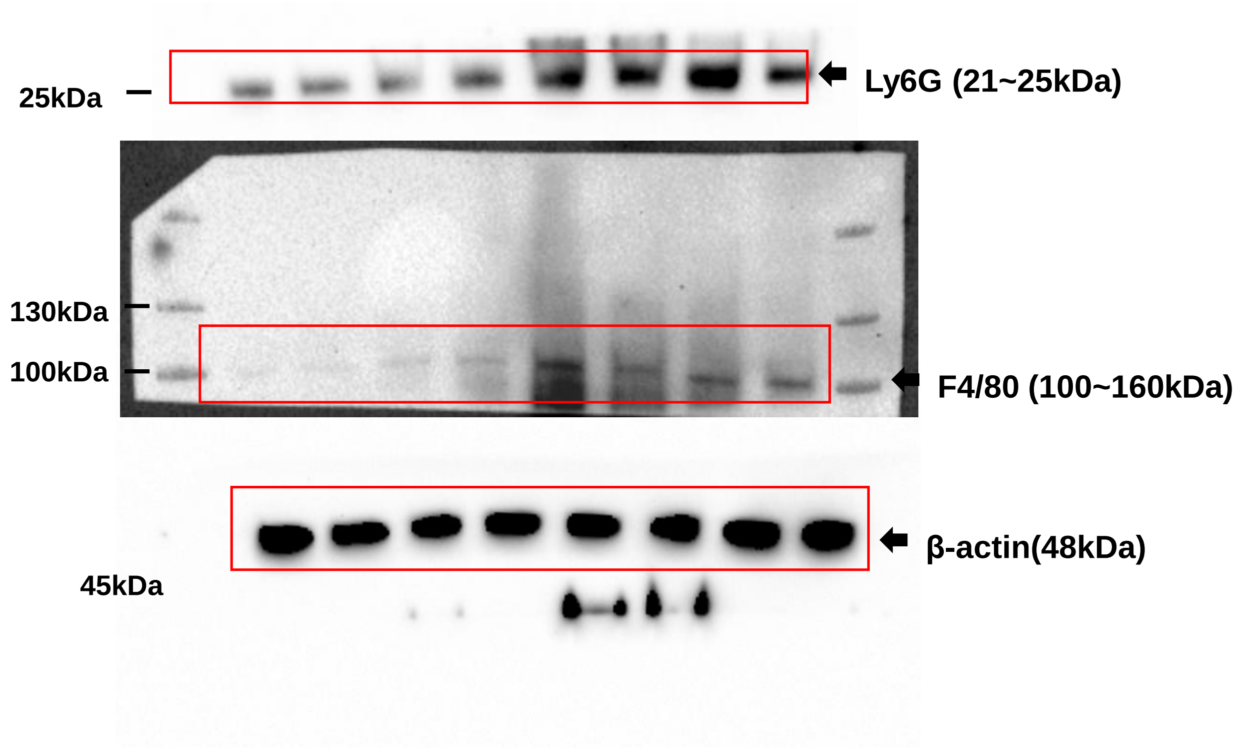

Without boxes

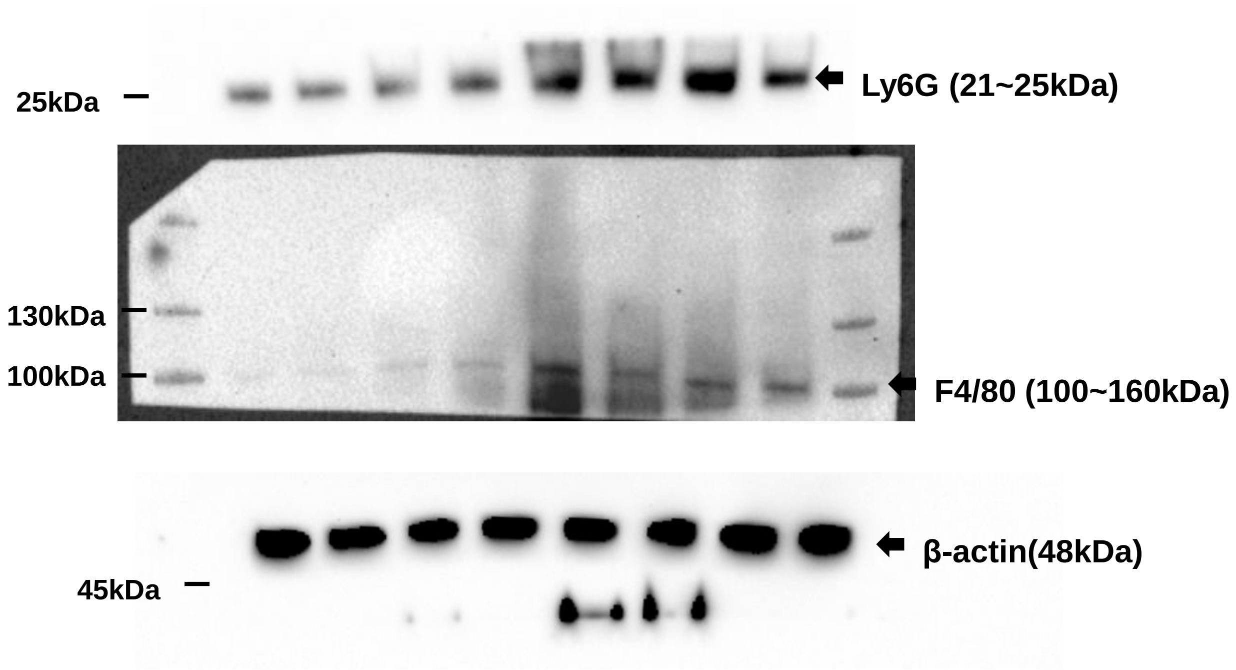

In order to efficiently use the limited samples and avoid the non-specific back ground, membranes were cut prior to hybridization with antibodies.
